
JOURNAL INFORMATION
==============================
NLM Title Abbreviation: ScientificWorldJournal
Journal ID: ScientificWorldJournal
NLM Journal ID: 101131163
Journal ID: TSWJ

The Scientific World Journal

ISSN: 2356-6140
EISSN: 1537-744X
Publisher: Wiley

ARTICLE INFORMATION
==============================
PMCID: PMC6084423
PMID: 30147596
DOI: 10.1100/tsw.2001.222
Article ID: 474617
Article version: 1
Subjects: Short Report

Fas-Induced Apoptosis of Human Neutrophils is Promoted by Phosphatidyl Inositol 3-Kinase but Suppressed by P38-MAPK Activity

Alvarado-Kristensson Maria
Poumlrn-Ares M. Isabella
Grethe Simone
Smith David
Zheng Limin
Andersson Tommy
Publication date: 2001
Electronic publication date: 2001 Oct 23
Volume: 1
First page: 116
Last page: 116
Copyright: Copyright © 2001 Maria Alvarado-Kristensson et al.
Copyright year: 2001
License: This is an open access article distributed under the Creative Commons Attribution License, which permits unrestricted use, distribution, and reproduction in any medium, provided the original work is properly cited.
License URL: https://creativecommons.org/licenses/by/3.0/

Funding: Swedish Cancer Association

==============================
Miami *Nature Biotechnology* Short Reports
*TheScientificWorld* (2001) 1 (S3), 116SR
ISSN 1532-2246; DOI 10.1100/tsw.2001.222

### FAS-INDUCED APOPTOSIS OF HUMAN NEUTROPHILS IS PROMOTED BY PHOSPHATIDYL INOSITOL 3-KINASE BUT SUPPRESSED BY P38-MAPK ACTIVITY

Maria Alvarado-Kristensson, M. Isabella Pörn-Ares, Simone Grethe, David Smith, Limin Zheng, and Tommy Andersson

Division of Experimental Pathology, Department of Laboratory Medicine, Lund University, U-MAS, S-205 02 Malmö, Sweden
Maria.Alvarado@exppat.mas.lu.se

**INTRODUCTION.** Mature neutrophils have a short life-span. The mechanisms involved in regulating neutrophil apoptosis are inadequately understood, although significant effects are exerted by factors in the local inflammatory environment. For instance, it has been reported that neutrophil apoptosis is delayed by lipopolysaccharide (LPS) and granulocyte-macrophage colony-stimulating factor (GM-CSF) but is accelerated by Fas ligand (FasL) (1). Neutrophil apoptosis is essential for resolution of inflammatory reactions (2), however only limited information exists regarding the involvement of protein phosphorylation events in the regulation of human neutrophil apoptosis. Therefore, we have studied the role of p38 MAPK and PI 3-K in Fas (CD 95)-mediated apoptosis.

**METHOD.** Neutrophils were isolated from whole blood (drawn from healthy volunteers). Apoptosis was measured by analysis of nuclear morphology (acridine orange and ethidium bromide staining), annexin V-binding (flow cytometry), and caspase-8 and –3 activity (fluorometric assay). p38 MAP Kinase activity was measured by in vitro assay (using ATF2 as a substrate) and by western blot studies of neutrophil cell lysates (using anti-active p38 polyclonal IgG). PI 3-K activity was determined in vitro (using phosphatidylinositol as a substrate) and by western blots showing accumulation of phosphatidylinositol 3-kinase p85 in the membrane fraction of the cells. Akt activation was determined by western blot (using anti-phospho Akt polyclonal IgG). We used western blot studies for showing Rac2 translocation to the membrane fraction of the cells.

**RESULTS AND DISCUSSION.** We discovered a previously undetected early and transient inhibition of the activity of p38 MAPK during neutrophil apoptosis.

Pharmacological inhibition of this enzyme (SB203580) augmented Fas-induced activation of caspases and the apoptotic response. The early inhibition of p38 MAPK was also concurrent with activation of PI 3-K, inhibition of which reduced apoptosis, thus supporting a role of PI3K as a proapoptotic-signaling molecule. This suggests that the p38 MAPK signals survival in neutrophils and that apoptosis is initiated during inhibition of this signal. This concept is strengthened by our findings that caspase-3 activity was initiated during the inhibition of p38 MAPK and that a transient inhibition of this kinase also accompanied spontaneous neutrophil apoptosis. Addition of GM-CSF abolished the transient inhibition of p38 MAPK activity, supporting the concept that the inhibition is a permissive event in apoptosis of isolated human neutrophils.

PI3K has mostly been implicated in protecting other cell systems from apoptosis through its downstream target Akt (3). In neutrophils, Akt is not activated upon Fas stimulation. In contrast, we find an increased accumulation of Rac2, another well-known PI3K target (4), in the membrane fraction of these cells.

We conclude that PI3K activity and the transient inhibition of p38 MAPK potentiates the apoptotic response in neutrophils and may be essential for triggering apoptosis in these cells. Furthermore, the PI3K effect might be mediated by its downstream target Rac and independently of Akt activity.

**ACKNOWLEDGEMENT.** This work was supported by the Swedish Cancer Association.

**REFERENCES.**

1. Ward, I., Dransfield, I., Chilvers, E.R., Haslett, I., and Rossi, A.G. (1999) Pharmacological manipulation of granulocyte apoptosis: potential therapeutic targets. Trends Pharmacol. Sci. 20, 503-509
2. Weiss, S.J. (1989) N. Engl. J. Med. 320, 365-376
3. Scheid, M.P. and Woodgett, J.R. (2000) Curr. Biol. 10, R191-R194
4. Scita, G., Tenca, P., Frittoli, E., Tocchetti, A., Innocenti, M., Giardina, G., and Di Fiore, P.P. (2000) EMBO J. 19, 2393-2398
